# Alpha-band sensory entrainment alters the duration of temporal windows in visual perception

**DOI:** 10.1038/s41598-018-29671-5

**Published:** 2018-08-07

**Authors:** Luca Ronconi, Niko A. Busch, David Melcher

**Affiliations:** 1 Center for Mind/Brain Sciences (CIMeC), University of Trento, Rovereto, Italy; 2 Institute of Psychology, University of Münster, Münster, Germany

## Abstract

The phase and frequency of neural oscillations in the alpha band (8–12 Hz) have been recently proposed as key parameters for the temporal resolution of visual perception. Here, we tested the possible causal links between these oscillatory features and temporal integration/segregation. The individual alpha frequency (IAF) peak as obtained from resting-state electroencephalography was used to set the frequency of sensory (audio-visual) entrainment for the lower (IAF − 2 Hz) and upper (IAF + 2 Hz) alpha. Entrainment at IAF ± 2 Hz was administered in the prestimulus interval to align oscillations to a faster or slower rhythm. We densely sampled in time the accuracy for integration/segregation by using identical stimuli with different instructions. The spectral peaks of performance fluctuations over time were found in the upper or lower alpha band for the IAF + 2 and IAF − 2 Hz entrainment, respectively, implying that faster entrainment resulted in faster behavioral fluctuations. Moreover, the entrainment frequency had opposite effects on temporal resolution: faster entrainment improved segregation while slower entrainment improved integration. Performance fluctuations were almost in anti-phase between the two tasks, such that highest integration performance coincided with lowest segregation performance. These findings provide evidence for a direct link between changes in the alpha band and the temporal resolution of perception.

## Introduction

Despite our subjective impression of a continuous and smooth reality, the continuous flow of information coming from the sensory world is not elaborated in an analog fashion. On the contrary, our brain samples information periodically by discretizing sensory inputs according to its hardwired rhythms within and across the different sensory modalities^1,2^. On the one hand, the capacity to combine information over time can be advantageous for accurate and precise percepts and actions of objects that tend to remain stable over time. However, temporal integration might reduce sensitivity to rapid changes in incoming sensory input due to a dynamic environment or our own actions. For example, temporal integration of motion signals might lead to misinterpreting a 180 degree change in direction as a reduction in speed. Likewise, long temporal integration windows would reduce the effectiveness of tactile feedback during active touch. Thus, sensory processing relies on a balance between temporal integration (to improve our perceptual interpretations at low temporal resolution) and segregation (sensitivity to change with high temporal resolution).

The brain may balance between these two needs through alternating between elaboration of input over time (integration) and sensitivity to new input (segregation), in a rhythmic way. The idea that perceptual processing depends on the rhythmic sampling of sensory information was initially introduce in seminal neurophysiological studies^3–5^ and later confirmed using electroencephalographical (EEG) recording in humans, which consistently show a relationship between oscillatory phase and sensitivity to new input^6–9^. Together, these findings argue that fluctuations in detection, reflecting sensitivity to new input, are related to alpha rhythms. The idea of a rhythmic process finds support also in nonhuman primates studies showing that spikes in sensory areas are more likely to occur at a specific phase of the local field potential oscillations relative to the opposite phase^10^.

Building on this idea, other studies have investigated the role of neural oscillations in the temporal resolution of visual perception, defined as whether two items in a sequence are perceived as separate, individual events or instead combined into a single unique percept^11–19^ (for reviews see^20,21^). The key idea is that if two stimuli fall into the same oscillatory cycle they are temporally bound into a single percept, while two stimuli falling into separate cycles are parsed into two unique temporal events. There is increasing M/EEG evidence for a role of the ongoing oscillatory phase, especially in the alpha (8–12 Hz) and theta (4–7 Hz) band, in tasks measuring whether two stimuli are integrated or segregated in time^11–13,18^. Another relevant feature of brain oscillations that seems to be important to determine whether two stimuli are integrated or segregated in time would be represented by the oscillatory frequency: in theory, a faster oscillation would result in higher sensitivity to change than a lower frequency oscillation. Consistent with this idea, it has been shown that individuals with faster prestimulus or resting-state alpha frequency exhibit better temporal segregation within^14^ and across sensory modalities^22,23^. Similarly, participants showed a faster peak alpha frequency on trials requiring rapid temporal segregation compared to other trials requiring temporal integration^19^. In sum, there is substantial evidence showing that temporal resolution is determined by the frequency and phase of neural oscillations.

To provide more causal evidence linking different aspects of the oscillatory brain activity to perception, one strategy is to apply visual and/or auditory stimulation at a specific frequency in order to entrain ongoing neural oscillations. This entrainment leads to resonance phenomena in neural and perceptual activity^12,17,24–27^. In a recent study, we showed that the alignment of the ongoing oscillations to a particular entrained rhythm can impact the temporal pattern of segregation of stimuli over time, and this occurred only within a certain frequency range that includes the alpha band (8–12 Hz)^17^, in agreement with previous M/EEG studies reviewed above.

Nonetheless, various aspects of the relationship between neural oscillations and temporal windows in perception remain unclear. In particular, in our previous study^17^ we found limited evidence concerning the role of the alpha frequency in determining the segregation/integration of stimuli over time, in contrast with previous studies^14,22,23^. One possibility is that variability in the endogenous alpha frequency in our study may have masked any effects of the entrainment during the task, an issue that could be overcome by adjusting the sensory entrainment as a function of the individual alpha frequency (IAF) peak. Furthermore, previous studies^14,17,22^ all had in common the use of quite similar variants of the two-flash fusion task, which measures whether two stimuli are perceived as a unique event or instead two separate events. One limit of a two-flash fusion design is that these studies do measure segregation (perception of two flashes) but do not directly measure temporal integration but, rather, infer integration from perception of a single flash. Indeed, perception of a single stimulus could result from temporal integration, as is usually assumed, but it might also result from failure to detect one of the two stimuli due to other factors such as lack of attention. In fact, many studies have implicated alpha rhythms in detection^7–9,28^, complicating the interpretation of “miss” trials: are they due to temporal integration or just a failure to detect both stimuli?

Ideally, it would be good to utilize a paradigm that measures both temporal integration (i.e. to achieve stability of object identity and location) and segregation (i.e. to support sensitivity to change) in combination with sensory entrainment, providing a method to measure whether there is a direct link between oscillatory dynamics and temporal aspects of perception. Moreover, considering recent evidence showing that power and frequency of neural oscillations are not completely independent parameters^29^, the use of sensory entrainment, which should entail a constant amplitude of the entrained oscillations, could be important in elucidating any link between the ongoing frequency and the temporal resolution of perception independently from power variations. Here, we aimed to clarify the role of the neural oscillations in determining the temporal windows of visual perception. We used multi-sensory (i.e. audio-visual) entrainment in the prestimulus period, followed by a temporal segregation/integration task that constituted a variant of the missing-element task^16,19,30–32^. This task had the advantage of employing an identical display sequence to measure temporal integration or segregation, with performance depending only on the specific task instruction. Entrainment was delivered at the upper and lower boundaries of the individually defined alpha band, by choosing for each subject the two stimulation frequencies based on the individual alpha frequency (IAF) peak obtained from the resting state EEG. In order to test the effect of entrainment on each participant, we presented stimulation at IAF–2 Hz for the lower alpha band and IAF + 2 Hz for the upper alpha band in order to see if we could also shift the frequency of behavioral oscillations. In addition, we tested the hypothesis that integration and segregation would be complementary, such that time periods in which one task was better the performance in the other task should be worse.

## Methods

### Participants

A total of 29 participants (12 males) aged 20–30 took part in the present study as paid volunteers. They reported no history of neurological disorders or epilepsy. All of them reported normal or corrected-to-normal vision and normal hearing, and gave informed written consent. The experimental protocol was approved by the University of Trento ethical committee and was conducted in accordance with the Declaration of Helsinki.

### Apparatus and stimuli

All visual stimuli were displayed on a 22.5′′ VIEWPixx monitor with a vertical refresh rate of 100 Hz. The auditory stimuli used for the entrainment were sinusoidal 500 Hz sounds presented through professional headphones. The visual stimulus used for the entrainment was a black square sized 7 × 7 degrees of visual angle, centered on the fixation point.

**Figure 1:**
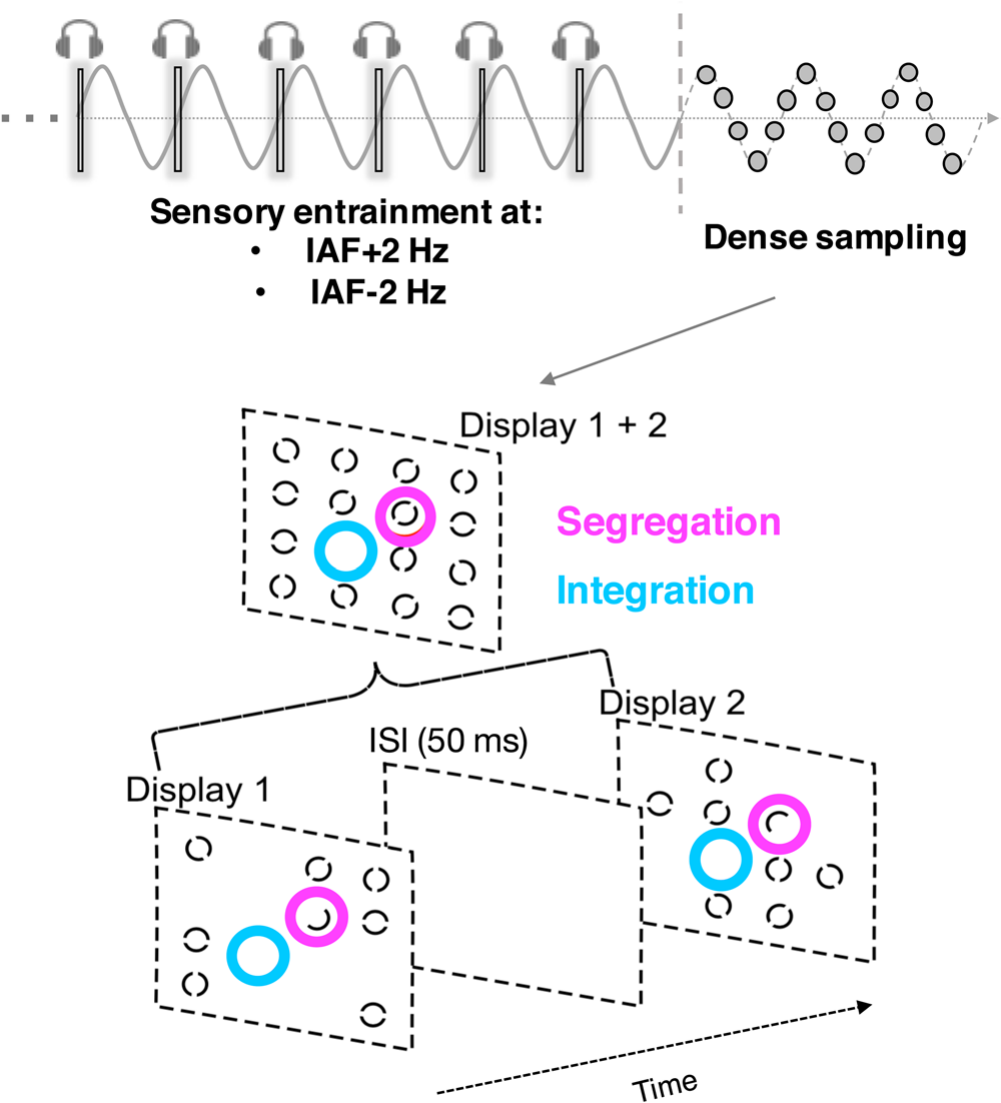
Experimental procedure. The individual alpha frequency (IAF) peak (see Methods and Fig. 2) was used to set the individualized entrainment frequencies (IAF ± 2 Hz). Sensory (audio-visual) entrainment at these two frequencies (randomized across trials) was delivered in the prestimulus period of a temporal integration/segregation task, where participants were asked to search and report the location of the odd element (i.e. the two half annuli complementing each other across displays) in segregation trials, and to search and report the location of the missing element (where there was a blank location with no annuli in the grid) in integration trials. The performance in this task was densely sampled in time at different delays after the entrainment offset to characterize the perceptual oscillations following the entrainment.

The stimulus for the main task was made up of two displays (Fig. 1) which were shown sequentially, separated by a blank interval, as a variant of the missing element task^16,19,30–32^. Critically, the target for the integration and for the segregation tasks were both shown on each trial, with only the task instruction that varied across blocks. The two target displays contained annuli placed within an invisible 4 × 4 quadratic element grid (each square was 1 × 1 deg). These stimuli were shown in two different and separated frames. Seven random locations (14 over both frames out of 16 total) were filled with a full black annulus on a uniform gray background (0.5 deg size, 0.06 deg line width; 0.5 deg space between grid locations). Thus, there was one missing location in the grid, which was the target for the integration task. Each annulus was split by a central gap with a randomly chosen orientation that could be 0°, 45°, 90°, or 135°. In addition, there was one “odd element” with a half annulus in each of the two displays, such that the two half annuli complemented each other across displays. This half annulus was the target on segregation trials. Each trial contained both a missing location (integration target) and a location in which only one half of the annulus was shown on each display (segregation target). The experiment was programmed in Matlab, using the Psychtoolbox^33^, and all visual stimuli were displayed on a middle grey background.

### Procedure

All trials started with the onset of a fixation point for 1000 ms, after which the entrainment period started. A combination of synchronized visual and auditory stimuli was used to maximize the possibility of yielding strong temporal entrainment^17,34,35^, since both visual and auditory onsets have been shown to induce a “phase reset” of neural oscillations within^36^ (i.e., from visual stimuli to visual areas) and also across^37,38^ sensory modalities (i.e., from auditory stimuli to the visual areas).

**Figure 2:**
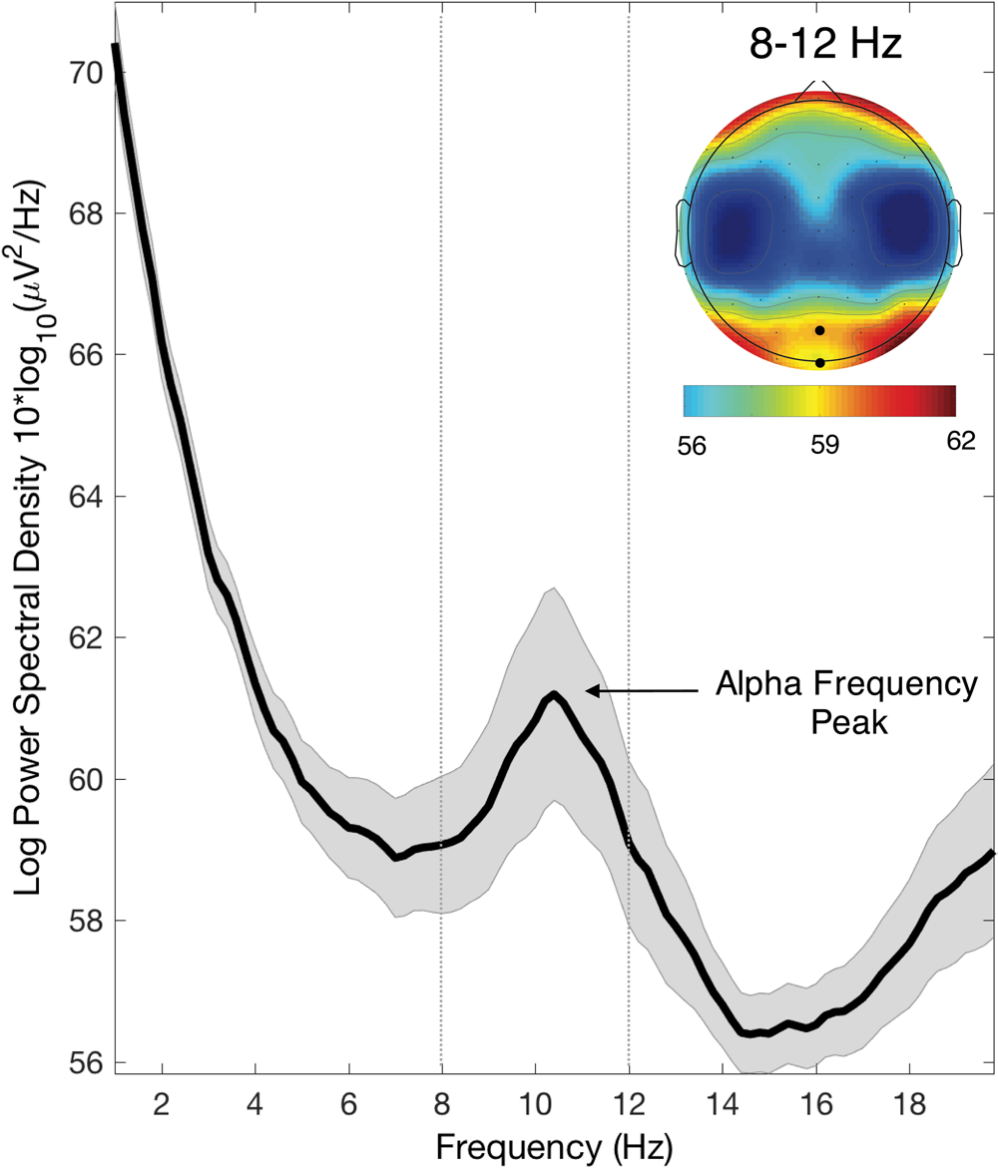
Individual alpha frequency (IAF) peak extraction. Power spectral density plot of the resting-state EEG averaged over participants (electrodes Oz and POz as highlighted in the topographical map), showing the usual peak in the alpha band (8–12 Hz) visible with higher power in posterior electrodes (shaded are represents the SEM). The EEG power spectrum of each participant was used to estimate the individual alpha frequency (IAF) peak.

Each audiovisual pair was looped 16 times before the onset of two target displays. The audiovisual entrainment stimuli were presented at a rate that was calculated based on the individual alpha frequency (IAF) as obtained from the resting state EEG for each participant (see next paragraph and Fig. 2). Due to limits in the refresh rate of the monitor (100 Hz), the IAF was rounded to the nearest integer number in the range between 8 and 12 Hz. In the ‘upper alpha condition’ the entrainment frequency was calculated as IAF + 2 Hz (the maximum possible entrainment frequency was ~14 Hz), while in the ‘lower alpha frequency’ the entrainment frequency was calculated as IAF − 2 Hz (the minimum possible entrainment frequency was ~6 Hz). For all entrainment frequencies, the duration of the audiovisual stimuli was set to 2 refresh cycles (20 ms). The time interval that separated the successive audiovisual stimulus presentation determined the precise entrainment frequencies used: in particular, the time interval between two successive audiovisual pair was 140, 120, 100, 90, 80, 70, 60 or 50 ms (resulting in a stimulus onset asynchrony or SOA of 160, 140, 120, 110, 100, 90, 80 or 70 ms) for entrainment frequency of 6.25, 7.14, 8.33, 9.09, 10, 11.11, 12.5 or 14.28 Hz, respectively. Consequently, the total duration of the entrainment period ranged between 1120 ms (14.28 Hz entrainment) and 2560 ms (6.25 Hz entrainment). The stimulus presentation methodology is illustrated in Fig. 1.

Performance in the temporal integration or segregation tasks was densely sampled in time by randomly varying the delay between the end of the entrainment sequence (our alignment point) and the presentation of the two target displays (the onset of the first target display, since the time interval between the first and the second target display was always fixed, see below). We followed the logic of previous studies that have demonstrated that it is possible to measure behavioral oscillations by carefully measuring fluctuations in behavior over time aligned to a putative reset point^17,36,37^. To sample the integration/segregation performance densely, we used 15 regularly spaced levels of delay between the end of the entrainment sequence and the presentation of the two target displays. The delays ranged between 20 and 300 ms (from 2 to 30 refresh cycles) in steps of 20 ms, so that we could cover three hypothetical alpha cycles at 10 Hz, a range within which previous studies showed the maximal effect for sensory entrainment of a similar duration^12,25,26^. In particular, Mathewson and colleagues found a significant increase in EEG phase locking across trials with 12 Hz rhythmic stimulation lasting for 200–300 ms after the onset of the entrainment^12^; similarly, Spaak and colleagues found similar results with 10 Hz visual stimulation using MEG, with effect lasting for at least 3 cycles (300 ms) after the end of the entrainment^26^. These two similar findings suggest that entrainment effects would continue for 2 to 3 alpha cycles after the offset of the entraining stimulus for entrainment sequences with a duration similar to the one employed here.

Each target display (containing the 7 circles, one blank location and one half-circle) was shown for 1 refresh cycle (10 ms) separated by a blank interval equal to five refresh cycles (50 ms). This value was chosen based on previous studies from our lab^16,19,32^ showing that this time interval was optimal to reach an intermediate accuracy level (across integration/segregation conditions) around 60–70% (chance level = 6.25%), which gave us reasonable margins to test the eventual performance fluctuations over time caused by the entrainment. Importantly, a 50 ms time interval fits within the alpha duty cycle^39^ (if we consider 10 Hz as the central alpha frequency), and thus is particularly suitable to test variation of performance in relationship to variation in the ongoing alpha phase and frequency.

In different blocks, participants were instructed to localize either the odd element or the empty location. Crucially, finding the odd element requires segregating the displays over time, whereas integration results in the perception of a full annulus, identical to the 14 other annuli. In contrast, finding the missing element (i.e. the empty location) in both displays requires integrating the displays over time, whereas segregation results in the perception of two separate displays with many empty locations. Critically, both the odd and missing elements were shown in all trials, so that integration and segregation were tested using the identical stimuli, only using different task instructions^16,19,32^.

At the end of each trial, participants were asked to report the correct location by clicking on the corresponding stimulus position in the grid, reporting the location of the odd element (half circle) in the segregation trials, and the location of the missing element in the integration trials. The response was given with no time constraints (Fig. 1). The total amount of trials administered for each participant was 900, consisting of 450 segregation trials and 450 integration trials (each of them containing 225 trials for both entrainment frequencies, thus 15 trials for each delay). The experiment was presented in 6 separate blocks of 150 trials (3 for segregation and 3 for integration), whose order was counterbalanced across subjects. The entire experimental session lasted 70–90 min.

### Resting-state EEG

The IAF peak used for the individual adjustment of the entrainment frequency was obtained in a separated session in which EEG data were recorded with a 64-channel system for AC/DC recording (Brain Products) with 62 cortical electrodes placed according to the international 10–20 system and 2 electrodes used for EOG monitoring. Resting data were obtained from 3 min of eyes-closed recording (online referenced to Cz). Offline, the data were referenced to an average reference, and band-pass filtered between 0.01 and 30 Hz. Data were visually inspected to remove data segments contaminated by muscular artefacts before extracting the FFT spectrum. The individual IAF peak was calculated as the frequency corresponding to the maximum FFT power values between 8 and 12 Hz, averaged across the channels POz and Oz. The average IAF across participants was 10.45 Hz (SD = 0.98 Hz) (Fig. 2).

### Data availability statement

A public data repository is available at the following link: https://osf.io/e27kc/#.

## Results

### Power spectrum of perceptual oscillations

**Figure 3:**
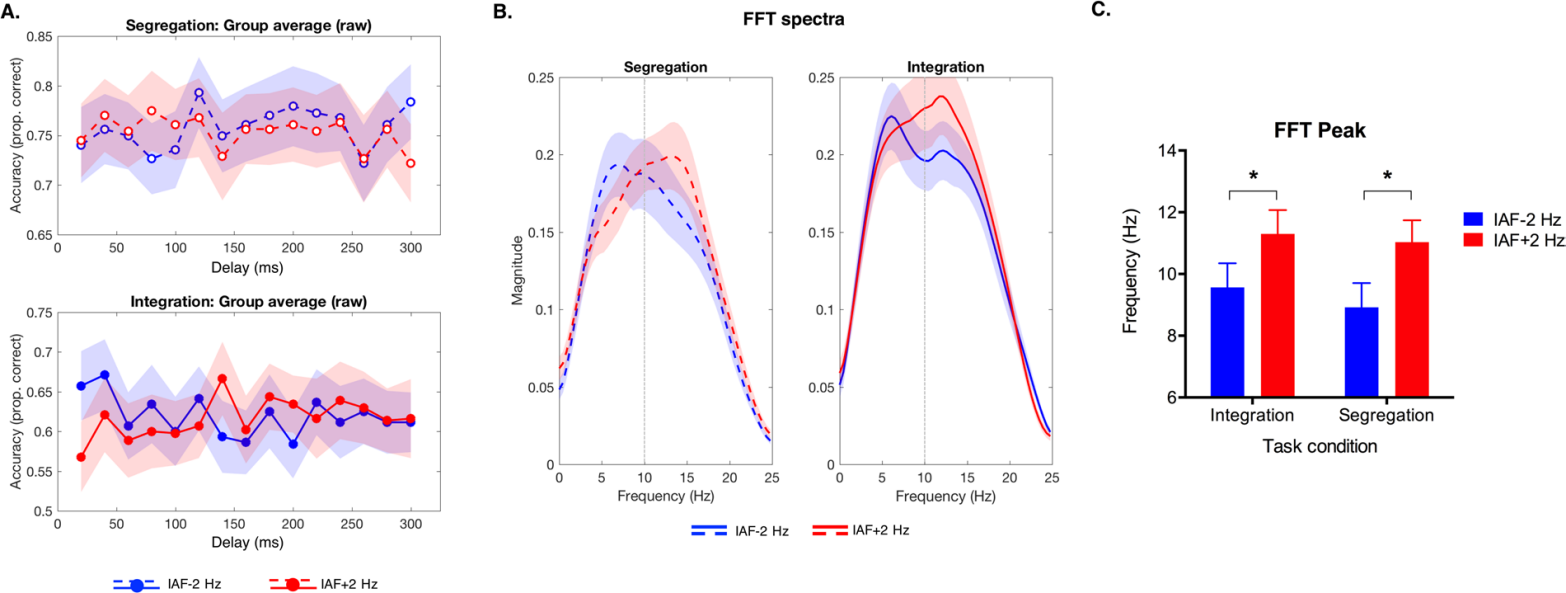
Power spectrum of perceptual oscillations induced by the entrainment. (**A**) Raw data were sorted for each participant as a function of the delay between the entrainment offset and the appearance of the first target display, and then preprocessed (see Methods) to calculate (**B**) the Fast Fourier Transformation (FFT) from which we extracted the power spectra of perceptual oscillation. In both (**A**) and (**B**) data were averaged across participants, with shaded area representing the SEM. (**C**) Plot showing the mean FFT peak across participants as a function of the entrainment frequency (IAF − 2 Hz vs. IAF + 2 Hz) and task condition (segregation vs. integration) (*p < 0.05, one tail t-test).

The aim of this analysis was to characterize the main oscillatory component of the perceptual oscillations following entrainment and to test differences across the experimental conditions. For each participant, raw data (Fig. 3A) from all trials were first sorted by the delay and de-trended. Subsequently, similarly to what has been done in other recent studies measuring behavioral oscillations^17,40,41^, a band-pass filter (IIR Butterworth filter, order 4) with a lower cutoff frequency of 2 Hz and higher cutoff frequency of 18 Hz was applied to the de-trended data. After the application of a Hamming window and zero padding, we performed the individual Fast Fourier Transform (FFT) for each combination of entrainment frequency (IAF + 2 Hz and IAF − 2 Hz) and task condition (integration and segregation). From the resulting FFT spectra (Fig. 3B), the frequencies corresponding to the maximum power (i.e. the peak of the behavioral oscillations power spectrum) observed in the frequency range of interest (2–18 Hz), was used as the dependent variable in a repeated-measure analysis of variance (ANOVA), where within-subjects factors were the ‘entrainment frequency’ (IAF + 2Hz vs. IAF − 2 Hz) and ‘task condition’ (integration vs. segregation). The ANOVA revealed a significant main effect of the entrainment frequency (F_(1,28)_ = 6.65, p = 0.015, η^2^ = 0.192), while the main effect of the task condition and the interaction were not significant (both Fs < 1). To further explore the effect of the significant main effect of the entrainment frequency, we performed one-tailed paired samples t-tests, which revealed that for both task conditions, the peak frequency was higher after the IAF + 2 Hz entrainment as compared to the IAF − 2 Hz entrainment (segregation: t_(28)_ = −1.84, p = 0.038; integration t_(28)_ = −1.98, p = 0.029; Fig. 3C), implying that faster entrainment resulted in faster fluctuations of performance.

This ANOVA on the FFT peaks revealed the presence of frequency-specific effects in behavioral oscillations, a result which is important to show that the perceptual oscillations in both tasks were driven by the preceding entrainment. A complementary approach to test the presence of significant behavioral oscillations involves permutation testing, as used in several other previous studies of perceptual oscillations^17,36,37,42^. This permutation approach typically involves keeping all preprocessing steps constant, but adds a step in which the delay of target onset time is randomly shuffled across trials. This makes it possible to calculate the power spectrum of behavioral oscillations under the null hypothesis in order to test the presence of significant peaks in the observed power spectrum, in particular by addressing which FFT magnitude values are above the 95th percentile as compared with the permuted distribution for each frequency point. We performed such permutation test, while keeping all the other pre-processing steps described above constant (i.e. detrending, filtering, Hamming-windowing and zero-padding). However, we found no significant peaks when comparing against the null distribution obtained with 10,000 random permutations of the real data. We are inclined to attribute this null result to the limited power we have in the present study, combined with the variability in the entrainment frequency. In terms of power, we had four conditions in a within-subject design, and this necessarily limited the amount of trials for each sampling point relative, for example, to our previous study^17^, in which there were only two entrainment frequencies in a between-subjects design, such that the number of trials per sampling point was three times higher. In addition, in our previous study we used a fixed entrainment frequency, per condition, rather than varying it across participants. Given that the permutation test compares power at each single frequency to the null distribution, dividing that power across several different frequencies dramatically reduces the power at any one frequency.

### Phase difference between oscillations in segregation and integration

The aim of this analysis was to test the presence of phase differences between integration and segregation that characterized the perceptual oscillations following the entrainment. To this aim we used the MATLAB CircStat toolbox^43^.

**Figure 4:**
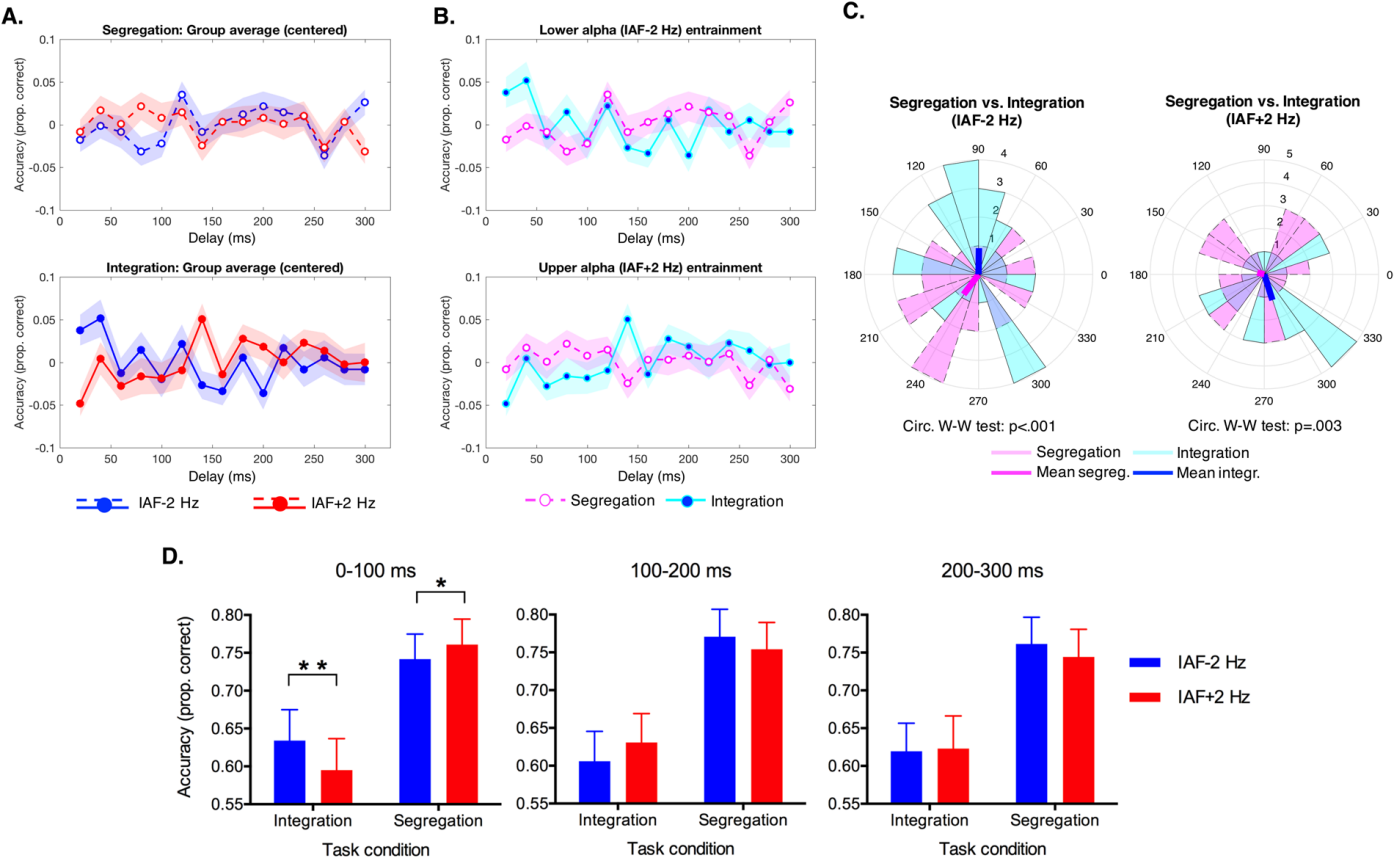
Phase differences between temporal integration and segregation and modulation of task accuracy as a function of the entrainment frequency. (**A**) Raw data centered on the mean for segregation (upper plot) and integration (lower plot) as a function of the entrainment frequency. (**B**) Raw data centered on the mean for IAF − 2 Hz entrainment (upper plot) and IAF + 2 Hz entrainment (lower plot) as a function of the task condition. In both (**A**) and (**B**) data were averaged across participants, with shaded area representing the SEM. (**C**) Phase distribution histogram of perceptual oscillations aligned to the IAF + 2 Hz (left plot) or IAF − 2 Hz (right plot) frequency, showing in both cases a significant phase difference between tasks as measured by the circular Watson-Williams test (ticker lines represent the mean phase angle across participants for integration or segregation). (**D**) Raw data averaged across participants (error bars represent the SEM) and in subsequent temporal bins (0–100 ms, 100–200 ms and 200–300 ms) as a function of entrainment frequency and task condition (*p < 0.05 and **p < 0.01, one tail t-test).

For each participant we extracted the phase angles in a frequency range that was centered on the frequency corresponding to the average peak of the power spectrum (specifically, we performed a circular average of phase angle data within a frequency range of 3 Hz centered on the FFT spectra power peaks plotted in Fig. 4C) for each of the four combinations of entrainment frequency and task condition. We decided to take the mean phase angle data from a small range around the FFT peaks for two main reasons: first because we did not use the same frequency of entrainment for all participants; second because even for the same entrainment frequency there are individual differences in the effect of the entrainment that we wanted to take into account. By including a small band around the peak, small variations in the individual FFT peaks that were present due to the reasons mentioned above would be expected to exert a smaller influence.

To test the phase difference between integration and segregation, we first checked that the two concentration parameters did not differ for the data (i.e. vectors of phase angle) of the comparison of interest. Since for both entrainment conditions no significant difference in phase concentration were found between integration and segregation (IAF − 2 Hz entrainment: F_(1,56)_ = 1.01, p = 0.97; IAF + 2 Hz entrainment: F_(1,56)_ = 1.17, p = 0.68), we then performed the parametric Watson-Williams test. For both entrainment conditions, we found a significant phase difference between integration and segregation (IAF − 2 Hz: F_(1,56)_ = 20.25, p < 0.001; IAF + 2 Hz: F_(1,56)_ = 9.86, p = 0.003) that was close to a phase opposition (180° phase difference): 143.8° for the IAF − 2 Hz entrainment condition and 122° for the IAF + 2 Hz entrainment condition. This phase difference between tasks was replicated with comparable results even when we used a bidirectional filter that ensured zero phase lag, and implies that moments yielding highest integration performance yielded lowest segregation performance, and vice versa, consistent with a previous report^16^.

### Task accuracy over time as a function of entrainment and temporal integration/segregation

Considering the evidence of oscillatory patterns in temporal perception induced by the entrainment as shown by the results described above, we then wanted to explore if the performance for the two types of task was effectively changed by the entrainment in the expected direction. According to the studies reviewed in the Introduction, we would expect faster ongoing alpha frequency to result in improvements on trials requiring temporal segregation, but a performance decline in trials requiring temporal integration. The opposite pattern should emerge with slower ongoing alpha frequency, which should lead to improvements on trials requiring temporal integration and performance decline in trials requiring temporal segregation.

To test this hypothesis, we first performed a repeated measures ANOVA on the accuracy rate (dependent variable) with ‘entrainment frequency’ (IAF + 2 Hz vs. IAF − 2 Hz) and ‘task condition’ (integration vs. segregation) as within-subjects factors, averaging the accuracy over all time points (delays) after the end of the entrainment. This ANOVA revealed only a main effect of the task condition (F_(1,28)_ = 6.41, p = 0.017, η^2^ = 0.186), with higher overall performance on segregation (mean = 0.76, SD = 0.18) as compared to integration trials (mean = 0.62, SD = 0.21). On the contrary, the main effect of the entrainment frequency (p = 0.312) and the interaction (p = 0.922) were not significant.

However, the effect of such a short entrainment (~1–2 sec) on peak alpha frequency is expected to be strongest immediately after the end of the rhythmic audio-visual sequence, as highlighted by previous neurophysiological evidence^26^. Thus, the performance sampled at later delays after the entrainment could be less affected by the effect of entrainment on oscillatory speed, and this seems to be confirmed when looking at the raw time course of accuracy data visible in Figs 3A and 4AB. To take into account this possible change of the entrainment strength over time, we repeated the same ANOVA on the accuracy rate described above with an additional factor ‘time windows’ (3 levels: 0–100 ms, 100–200 ms and 200–300 ms), according to which accuracy was averaged across the delays within these three time windows corresponding to the three hypothetical alpha cycles after the entrainment. Beyond the significant main effect of task condition described above, this ANOVA revealed a significant three-way interaction task condition*entrainment frequency*time windows (F_(2,56)_ = 9.7, p < 0.001, η^2^ = 0.257; Fig. 4D). When looking at post-hoc ANOVAs performed within each of this time window, we found a significant two-way interaction task condition*entrainment frequency in the time window 0–100 ms (F_(1,28)_ = 11.51, p = 0.002, η^2^ = 0.291) and 100–200 ms (F_(1,28)_ = 8.07, p = 0.008, η^2^ = 0.224), but not in the third last time window between 200–300 ms (p = 0.321). Planned comparisons (one-tailed paired-sample t-tests: segregation IAF + 2_Hz_ > segregation_IAF − 2Hz_; integration_IAF + 2Hz_ < integration_IAF − 2Hz_) showed, as expected, that in the time window 0–100 accuracy in segregation trials was higher for the IAF + 2 Hz entrainment (mean proportion correct = 0.76, SD = 0.18) relative to the IAF − 2 Hz entrainment (mean = 0.74, SD = 0.18; t_(28)_ = −2, p = 0.028), while accuracy in integration trials was higher for the IAF − 2 Hz entrainment (mean = 0.63, SD = 0.22) relative to the IAF + 2 Hz entrainment (mean = 0.59, SD = 0.22; t_(28)_ = 2.7, p = 0.006). In the time window 100–200 ms, no significant planned comparisons emerged in the hypothesized direction (both p > 0.94).

## Discussion

Recently, a growing body of evidence coming from M/EEG studies has provided support for the idea that both the phase^11,12,15,18^ and frequency^14,19,22^ of the ongoing (prestimulus) oscillations in the alpha band are closely related to the temporal mechanisms of integration and segregation in visual perception. In the present study, we wanted to test a causal link between phase and frequency of neural oscillations in the alpha band and temporal mechanisms of visual perception by entraining neural oscillations in the pre-stimulus period^17,24–26,34,35^. We used short (~1–2 sec) multi-sensory entrainment in the prestimulus period to attempt to align the ongoing neural oscillation to a slower or faster alpha rhythm. We chose the precise entrainment frequency for lower and upper alpha band stimulation based on the individual alpha rhythm (IAF peak) as determined from a resting-state EEG. As a probe for temporal segregation and integration we used a variant of the missing-dot task in which an identical sequence of displays was used to measure temporal integration or segregation according to the specific task instruction. The accuracy in this task was densely-sampled in time (i.e. 50 Hz sampling frequency) to reveal any oscillatory pattern that might emerge as a consequence of the entrainment.

Our results show that the entrained rhythm resonated with the rhythmic fluctuations of behavioral performance at the same frequency. Moreover, the frequency of the behavioral oscillations followed a comparable rhythm for temporal integration and segregation, just out of phase for the two tasks. Faster entrainment frequencies (IAF + 2 Hz) led to faster fluctuations in behavioral performance for both temporal integration and segregation, and the opposite emerged for lower entrainment frequencies (IAF + 2 Hz), which led to slower fluctuations in performance. This result is in line with recent evidence showing the flexibility of temporal windows in perception, which can be modulated both by transcranial alternate current stimulation and sensory entrainment^17,22^. They also confirm the increasing evidence showing a link between the phase of the ongoing oscillations and the temporal organization of visual perception. Using a task that allows us to clearly disentangle the two sides of temporal perception (integration versus segregation), the present results give stronger support to the idea that the phase of ongoing neural oscillations determines both temporal integration and segregation, and that the relationship between phase and temporal perception emerges also in paradigms that do not infer integration from perception of a single flash in a two-flash fusion design (as used in different variants in many previous studies)^14,15,17,18^, which by itself could potentially be affected by sensitivity issues (e.g. failure to detect one of the two stimuli). It is important to note that the final stimulus in the audiovisual entrainment sequence was matched across the two different IAF +/− 2 entrainment conditions in terms of duration and temporal distance from the test (integration/segregation) stimuli. Thus, any affect of forward masking should be similar across the two entrainment frequency conditions. Interestingly, spatial attention leads to a similar increase in performance for both integration and segregation, and over a period of time much longer than that tested here^32^, meaning that any changes in the salience of the two entrainment stimuli might lead to a general increase or decrease in performance but would not predict the pattern of fluctuations in performance over time, in counter-phase for the two tasks.

Concerning the analyses of phase difference between perceptual oscillations in integration and segregation aligned to a specific entrainment frequency, our results suggest that the two sides of temporal perception manifest as two nearly opposing time courses. Indeed, for both entrainment frequencies we found a significant phase difference with values that were close to full phase opposition (180° difference) between temporal integration and segregation. This confirm previous findings showing the presence of phase opposition between segregation and integration in behavioral and MEG data^16^. In particular, a pattern of similar results was obtained in a previous study by Wutz and colleagues^16^, which employed a behavioral dense sampling procedure similar to the one employed here, where the alignment point was not the entrainment offset but onset of a new eye fixation. Our results reinforce the idea that temporal perception is characterized by opposite needs that balance each other over time, with temporal integration aiming at stability of perceptual representation over time, and segregation aiming to provide the maximum sensitivity to changes in the information flow. These results are consistent with the idea that brain states alternate between periods of (a) increased sensitivity to external stimulation and (b) the full and recurrent processing of information already present in the brain.

A last important aspect emerging from our study is the opposite change in performance that was observed immediately after the entrainment (within 100 ms) as a function of the entrainment frequency. Specifically, we observed a significant improvement in segregation accuracy after the entrainment at the upper alpha band (IAF + 2 Hz) as compared to the entrainment at the lower alpha band (IAF − 2 Hz). The opposite was true for integration trials, where accuracy increased after the entrainment at the lower as compared to the upper alpha band. These results provide direct evidence that the peak frequency of the ongoing oscillation has a role in determining the temporal resolution of perception, as proposed by recent M/EEG studies^14,19,22,23^, with opposite effects of increasing or decreasing the ongoing frequency on segregation and integration.

Using sensory entrainment at different frequencies should ensure that any amplitude/power variations of the ongoing oscillations should not have influenced these results, an aspect which is particularly relevant considering recent findings by Nelli and others^29^ showing that instantaneous frequency and amplitude of alpha oscillation are not completely independent mechanisms. Although our study could not be exhaustive in addressing this question, since only by having M/EEG recording during the data collection would it be possible to confirm that alpha frequency differed in the two entrainment conditions without any change in amplitude, it is useful to consider a previous study by Mathewson and colleagues^12^. Those authors compared 12 Hz rhythmic visual entrainment to a control, non-rhythmic, stimulation condition and showed that after the end of the entrainment there were significant differences in phase locking of alpha oscillations but no significant differences in power between the rhythmic entrainment and the control (non-rhythmic) condition. Since the entrainment conditions we employed in the present study were both rhythmic, and thus more similar to each other than the conditions in the study by Mathewson and colleagues, we should not expect any significant power difference between IAF + 2 and IAF − 2 Hz. Another noteworthy aspect is that, differently from the previous study in which we used a two-flash fusion task with stimuli that were supra-threshold but at lower contrast, here in order to test both segregation and integration, we used complex and higher contrast stimuli and we cannot completely exclude that the first target display could have diminished the impact of the entrainment by causing a phase reset of the manipulated alpha frequency. Nonetheless, the effectiveness of our manipulation is reflected by the significant differences in the peaks of the FFT spectra.

In conclusion, the pattern of results reported here are consistent with the proposal that the alpha rhythm is closely involved in the temporal resolution of visual perception. The use of individually targeted sensory entrainment with two different tasks allowed us to test specific hypotheses in favor of this idea, while also controlling for some potential confounds from other studies. These results indicate that fluctuations towards temporal integration versus segregation are not simply a result of oscillations in detection of the stimuli. The dramatic changes in perceptual oscillations from such a brief period of sensory entrainment demonstrate some plasticity in the system and hold promise for more long-lasting entrainment paradigms to effectively speed up or slow down visual processing.
